# Polymorphism of Drug Transporters, Rather Than Metabolizing Enzymes, Conditions the Pharmacokinetics of Rasagiline

**DOI:** 10.3390/pharmaceutics14102001

**Published:** 2022-09-21

**Authors:** Pablo Zubiaur, Miriam Matas, Samuel Martín-Vílchez, Paula Soria-Chacartegui, Gonzalo Villapalos-García, Laura Figueiredo-Tor, Sofía Calleja, Marcos Navares-Gómez, Alejandro de Miguel, Jesús Novalbos, Gina Mejía-Abril, Sergio Luquero-Bueno, Manuel Román, Dolores Ochoa, Francisco Abad-Santos

**Affiliations:** 1 Clinical Pharmacology Department, Hospital Universitario de La Princesa, Instituto Teófilo Hernando, Universidad Autónoma de Madrid (UAM), Instituto de Investigación Sanitaria La Princesa (IP), 28006 Madrid, Spain; 2 Centro de Investigación Biomédica en Red de Enfermedades Hepáticas y Digestivas (CIBERehd), Instituto de Salud Carlos III, 28006 Madrid, Spain

**Keywords:** rasagiline, Parkinson’s disease, pharmacogenetics, pharmacokinetics, precision medicine

## Abstract

Rasagiline is a selective and irreversible inhibitor of monoamine oxidase type B with neuroprotective effect, indicated for the management of Parkinson’s disease. The aim of this work was to evaluate the impact of seven *CYP1A2* alleles and of 120 additional variants located in other CYP enzymes (e.g., *CYP2C19*), UGT enzymes (e.g., *UGT1A1*) or other enzymes (e.g., *NAT2*), and transporters (e.g., *SLCO1B1*) on the pharmacokinetic variability and safety of rasagiline. A total of 118 healthy volunteers enrolled in four bioequivalence clinical trials consented to participate in this pharmacogenetic study. *CYP1A2* alleles were not associated with the pharmacokinetic variability of rasagiline. Patients with *ABCB1* rs1045642 G/A+A/A genotypes presented higher area under the curve adjusted by dose per weight (AUC_0-∞_/DW) than those with the G/G genotype (*p* = 0.012) and lower volume of distribution (V_d_/F) and clearance (Cl/F) (*p* = 0.001 and *p* = 0.012, respectively). Subjects with the *ABCC2* rs2273697 A/A genotype presented lower t_max_ (i.e., the time to reach the maximum concentration, C_max_) compared to those with G/G+G/A genotypes (*p* = 0.001). Volunteers with the *SLC22A1* *1/*5 genotype exhibited lower C_max_/DW and higher t_max_ (*p* = 0.003 and *p* = 0.018, respectively) than subjects with the *1/*1 diplotype. Only one adverse drug reaction was reported: headache. Our results suggest the genetic polymorphism of drug transporters, rather than metabolizing enzymes, conditions the pharmacokinetics of rasagiline.

## 1. Introduction

Parkinson’s disease (PD) is the second most common chronic progressive neurodegenerative disorder in the world after Alzheimer’s disease (AD) [1]. It is characterized by the loss of dopaminergic neurons in the substantia nigra pars compacta (SNc) resulting in a dopamine deficit in the striatum that causes an alteration of the physiology of the basal ganglia, responsible for motor-related functions. Despite not being curative, there are different pharmacological treatments to control motor and non-motor symptomatology [1]. Levodopa is a dopamine precursor that is used as the main treatment to treat motor symptoms in the early stages of the disease [2]. Other therapeutic agents include monoamine oxidase (MAO) inhibitors, such as rasagiline, catechol-O-methyltransferase (COMT) inhibitors, such as entacapone, and dopaminergic agonists, such as ropinirole [1,2]. 

Rasagiline is a selective and irreversible monoamine oxidase type B (MAO-B) inhibitor, a mitochondrial enzyme that metabolizes catecholamines, including dopamine. Thus, rasagiline inhibition of MAO-B causes an increase in the concentration of dopamine in the striatum [3]. In addition, it displays a neuroprotective effect by (a) reducing the activation of caspase 3, and therefore of the generation of superoxide, a reactive oxygen species (ROS) that triggers cell apoptosis and (b) by increasing the intracellular level of glutathione [4]. It is indicated in monotherapy in patients with early PD and in combination therapy with levodopa when the disease is more advanced [5]. Among the most frequent adverse drug reactions (ADRs) are headache, depression, dyskinesia, orthostatic hypotension, and constipation [6]. These ADRs can affect patient adherence to treatment and, consequently, ineffectiveness of therapy, exacerbation of symptoms and reduction of their quality of life. MAO-B inhibition selectivity diminishes in a dose-dependent manner, and some ADRs, including weight loss, postural hypotension, and dry mouth, are consistently dose-dependent [7]. 

Rasagiline is administered orally in tablet form and the recommended dose is 1 mg per day [6]. It presents linear pharmacokinetics and rapid absorption, reaching the maximum plasma concentration (C_max_) at approximately 0.5 h (t_max_). Furthermore, it undergoes intense hepatic first-pass metabolism, with an oral bioavailability of 36%. Rasagiline shows a volume of distribution (V_d_) that can vary between 87 and 243 L, a plasma protein binding of about 60–70% and a high capacity to cross the blood-brain barrier [5,6]. Its biotransformation occurs almost completely in the liver before excretion. It is metabolized by N-dealkylation and/or hydroxylation forming mainly the metabolite S1-1-aminoindane which is not a MAO-B inhibitor [6]. In vitro studies suggest that rasagiline’s main metabolizing enzyme is the cytochrome P450 (CYP) 1A2 (CYP1A2) isoform. Urine elimination is predominant (62.6%). In addition, it has an elimination half-life (t_1/2_) of 0.6–2 h [6].

Scarce pharmacogenetic research on rasagiline has been published to date. A recent paper evaluated the impact of the *CYP1A2* -163C>A variant on rasagiline pharmacokinetics [8]. However, this variant appears in several alleles and the impact of each allele or haplotype may be different. Another paper reported that the dopamine D2 receptor (*DRD2*) gene rs2283265 and rs1076560 polymorphisms were predictors of treatment response [9]. In this work, we aimed to analyze the impact of the seven most prevalent *CYP1A2* alleles on rasagiline pharmacokinetic variability and safety. In addition, we aimed to explore the impact of several variants in other relevant pharmacogenes (including other metabolizing enzymes, transporters, etc.) and the pharmacokinetic variability of rasagiline and the incidence of ADRs. To our knowledge, this is the first work to provide such complete coverage of genes and variants in relation to rasagiline. The present work is part of the La Princesa Multidisciplinary Initiative for the Implementation of Pharmacogenetics (PriME-PGx) [10].

## 2. Materials and Methods

### 2.1. Study Design and Procedures

The data used in this work were obtained from four bioequivalence clinical trials conducted at the Clinical Trials Unit of the Hospital Universitario de La Princesa (UECHUP) (EUDRA-CT IDs: 2012-004433-17, 2013-002159-14, 2014-001123-77 and 2021-000315-23). All were open-label, crossover, randomized studies of two formulations of rasagiline after administration of a single oral dose to fasting healthy volunteers. The reference formulation (R) in all cases was Azilect^®^ 1 mg tablets (TEVA Pharmaceuticals Europe B.V.), which was compared against different test formulations (T), all of them 1 mg tablets, except in clinical trial 3, whose test formulation was orodispersible 1 mg. The four bioequivalence trials had two periods and two sequences (TR and RT). In the first period, the volunteers randomly were administered one of the two formulations and, in the following period, after a 7-day washout period, the opposite formulation. All of them were reviewed and approved by Independent Ethics Board (IEB) of the Hospital Universitario de La Princesa, and by the Spanish Drug Agency (AEMPS). In addition, they were performed in accordance with the guidelines of the International Conference on Harmonization for Good Clinical Practice (ICH-GCP), current Spanish legislation and the Revised Declaration of Helsinki [11]. The pharmacogenetic study (SFC-FG-2020-1) was likewise approved by the IEB of Hospital Universitario de La Princesa (registry number 4176, 9 July 2020).

Inclusion criteria included: women or men between 18 and 55 years of age, without organic or psychological disorders and without abnormalities in laboratory tests, physical examination, and medical history. Exclusion criteria included: subjects with body mass index (BMI) outside the range 18.5–30 kg/m^2^, pregnant or breastfeeding women, smokers, having participated in other clinical trials in the previous 3 months, history of sensitivity to any drug, having received any medication two days before receiving the study drug, and inability to collaborate and respect the instructions. A total of 174 volunteers participated in the clinical trials, 118 of whom gave informed consent to participate in the observational pharmacogenetic study.

In each period, the drug was administered under fasting conditions, which were maintained until 5 h after drug administration. Each subject provided 20 or 21 blood samples (depending on the clinical trial) that were extracted by BD Vacutainer^®^ vacuum system with EDTA K2 tubes for analysis at different times between 0 h (before receiving the drug) and up to 12 h after drug intake. Samples were centrifuged for 10 min at 1900× *g* and stored at −20 °C (±5 °C), until their shipment to the external analytical laboratory for quantification of plasma rasagiline concentrations. A high-performance liquid chromatography coupled to triple quadrupole mass spectrometry (HPLC-MS/MS) analytical method was used. The latter was validated according to European regulations for bioequivalence assessment [12], with a lower limit of quantification (LLOQ) of 10 pg/mL and a calibration range of 10–10,000 pg/mL.

### 2.2. Pharmacokinetics and Safety 

Pharmacokinetic parameters were calculated with WinNonlin Professional (Scientific Consulting, Inc., Cary, NC, USA) following a non-compartmental model. The area under the curve (AUC_0-t_), was obtained by the trapezoidal method. The extrapolation to infinity (AUC_0-∞_) was determined by the sum of two partial AUCs: AUC_t-∞_ calculated by the ratio Ct/k, where Ct is the last detectable concentration and k the elimination slope; plus AUC_0-t_. C_max_ and t_max_ were directly obtained from the plasma time-concentration curves. t_1/2_ was calculated as Ln2/k. Plasma clearance adjusted for bioavailability (Cl/F) was calculated as dose/AUC. Finally, the volume of distribution adjusted for bioavailability (V_d_/F) was calculated by dividing Cl/F by k. Clinical trial 3 formulations were not bioequivalent; consequently, only the pharmacokinetic parameters of Azilect^®^ from all the clinical trials were used for the pharmacogenetics study.

Rasagiline tolerability was addressed through the identification of adverse events (AEs), which was performed by asking the volunteers a generic question or by spontaneous notification. The causality between AEs and rasagiline intake was addressed with the Spanish Pharmacovigilance System algorithm [13]. For this work, only those AEs with a demonstrated causality relationship (i.e., definite, probable, or possible) were considered adverse drug reactions (ADRs). 

### 2.3. Genotyping and Phenotyping

One of the EDTA-K2 tubes extracted for the determination of plasma levels was reserved. The formed elements (i.e., the tubes after plasma extraction) were reconstituted with 0.9% sodium chloride solution and 500 µL were used for DNA extraction with an automated Maxwell^®^ RSC instrument (Promega, Waltham, MA, USA). A Qubit^®^ 3.0 fluorometer (ThermoFisher, Waltham, MA, USA) was used to measure DNA concentration, which was homogenized at 30–70 ng/ul. A QuantStudio 12K Flex qPCR instrument (Applied Biosystems, ThermoFisher, USA) with an Open Array thermal block and a customized array were used (Very Important Pharmacogene Open Array, v2, VIPOA2). This array, designed by the researchers of Clinical Pharmacology Department, Hospital Universitario de La Princesa, covers the most relevant polymorphisms in clinically relevant pharmacogenes. The Supplementary Table S1 shows the 120 variants and 33 genes analyzed for the present work. They were included for their potential relationship with rasagiline’s pharmacokinetic and safety variability.

The pharmacogenetic phenotype was inferred according to the Clinical Pharmacogenetics Implementation Consortium (CPIC) or Dutch Pharmacogenetics Working Group (DPWG) guidelines when available: *ABCG2*, *SLCO1B1*, *CYP2C9* [14]; *CYP2B6* [15]; *CYP2C19* [16]; *CYP2D6* [17]; *CYP3A4* [18]; *CYP3A5* [19]; *UGT1A1* [20,21]. For *NAT2*, the phenotypes (rapid or slow acetylators, RA or SA) were inferred as indicated previously [22]. Concerning *CYP1A2*, no phenotype was reported to date. Due to the complexity of *CYP1A2* allele definitions, and the relevance of this gene regarding rasagiline metabolism, an exhaustive haplotype analysis was performed to properly define them. Table 1 shows an allele definition table based on the presence or absence of the variants genotyped in the present work. The design of this table was based on PharmVAR entry for *CYP1A2* (available at: https://www.pharmvar.org/gene/CYP1A2, accessed on 1 September 2022) [23,24]. Briefly, *1A allele was assigned when the six genotyped variants were not present. Some of the alleles were inferred unambiguously (e.g., one individual with the -3860G>A A/A genotype and with any other variant present was considered *1C/*1C). To resolve alleles that cannot be defined unambiguously (e.g., one individual with 739T>G T/G and 5347T>C T/C genotypes could be *1B/*1E if the variants are located in “trans”, or in different alleles; or *1A/*1G if variants are located in “cis”, or in the same allele), prevalence data from the 1000 Genomes Project were used with the LDhapTool (available at: https://ldlink.nci.nih.gov/?tab=ldhap, accessed on 1 September 2022). 

*CYP1A1* and *CYP2C8* genes were analyzed as diplotypes. The remaining variants were analyzed individually because haplotypes or alleles are not properly defined nowadays: *ABCB1, ABCC2, ABCC3, CES1, COMT, CYP1B1, CYP2A6, CYP3A43, SLC22A1, SLC22A2, SLC28A3, SLC6A2, UGT1A3-4, UGT1A4, UGT1A6, UGT1A8, UGT1A9, UGT2B10, UGT2B15* and *UGT2B7*.

### 2.4. Statistical Analysis

The SPSS software (v23.0, SPSS Inc., Chicago, IL, USA) was used for statistical analyses. AUC0-∞, and C_max_ were divided by the dose/weight (DW) ratio to correct sex-related variability related to a varying weight. For the same purpose, V_d_/F and Cl/F were divided by weight. With the purpose of normalizing their distributions, all pharmacokinetic parameters were logarithmically transformed for statistical analysis. Initially, a univariate analysis was conducted of the demographic characteristics according to sex and ethnicity, and of the pharmacokinetic parameters or ADR incidence based on sex, biogeographical group (hereinafter, “ethnicity”), genotypes and phenotypes. *T*-tests were used for the comparison of means for variables with two categories and ANOVA tests for variables with three or more categories, followed by a Bonferroni post-hoc. The Shapiro–Wilk test was used to address the normality of demographic characteristics. For those not following a normal distribution, the nonparametric Kruskal–Wallis test was used. For variables following normal distributions, means and standard deviations (SD) were provided and for those not following normal distributions, the interquartile range (IQR, or the difference between the third and first quartile) was shown. Covariates (i.e., sex, ethnicity, and the clinical trial) and those factors reaching the statistical significance threshold (*p* < 0.05) were introduced as independent variables in a multivariate analysis, which was performed by regression. As dependent variables, all pharmacokinetic parameters were explored (linear regression) or each ADR (logistic regression). The Bonferroni correction for multiple comparisons was implemented to correct type-1 error. This correction was performed to semi-quantitatively weight the strength of the associations; significant associations after correction should not necessarily be considered clinically relevant due to the observational nature of the study. The following significance indicators were provided: the p value for univariate analyses; and the p value, the unstandardized beta coefficient (β) (linear regression) or the odds ratio (OR)/estimate (logistic regression) and the R^2^ determination coefficient for multivariate analyses. For associations reaching *p* < 0.05 in both univariate and multivariate analyses, the following structure was followed: p_univariate_; β or OR, R^2^, p_multivariate_. Generalized linear models (GLM) were subsequently performed to detect interactions between all the genetic variables showing significant associations (*p* < 0.05 after the correction for multiple comparisons) with any pharmacokinetic parameter (more details are included in the results section). 

## 3. Results

The participants of the study were 65 women and 53 men (total *n* = 118), of similar age, around 28 years old. Men presented greater height, weight, and body mass index (BMI) than women (*p* < 0.001, *p* < 0.001 and *p* = 0.008, respectively) (Table 2). Volunteers mainly self-reported as Caucasian (*n* = 83) whereas 34 self-reported as Latino-Americans and 1 as Black, who was merged with the Latino-American volunteers in a group called “Mixed” ethnicity. The group of Mixed ethnicity showed a higher age and BMI and lower height (*p* < 0.001, *p* < 0.001 and *p* = 0.003, respectively).

AUC_0-∞_ was significantly higher in women than in men (4.29 ± 1.41 h*ng/mL and 3.71 ± 0.88 h*ng/mL, *p* = 0.021). After DW correction, the differences disappeared; differences were observed in C_max_/DW, the latter being higher in men than women (*p* = 0.015; β = 0.173, R^2^= 0.157, *p* = 0.026), while t_1/2_ and V_d_/FW were lower in men (*p* < 0.001; β = −0.286, R^2^ = 0.163, *p* < 0.001; and *p* < 0.001; β = −0.327, R^2^ = 0.352, *p* < 0.001, respectively) (Table 3). In addition, Caucasians presented lower t_1/2_ than mixed ethnicity individuals (*p* = 0.040) (Table 3).

Rasagiline pharmacokinetic parameters based on *CYP1A2* diplotypes are shown in Table 4. No significant associations were observed. The most prevalent allele was *1F (53%), the most prevalent diplotypes were *1F/*1F (33%) and *1B/*1F (31%); no volunteer with the *1A allele was identified. No significant association was observed between diplotypes and pharmacokinetic parameters variability (Table 4).

**Figure 1:**
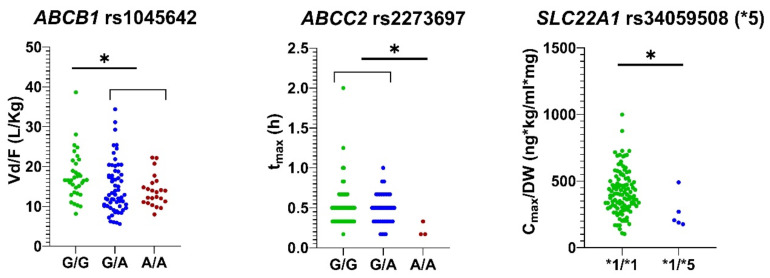
Box plots representing significant associations between genotypes and pharmacokinetic parameters after Bonferroni correction for multiple comparisons. **Left** panel: Vd/F according to *ABCB1* rs1045642 genotype; center panel: tmax according to *ABCC2* rs2273697 genotype; **right** panel: C_max_/DW according to *SLC22A1* rs34059508 genotype (*1/*1 equals to G/G, *1/*5 equals to G/A). * *p* < 0.001 after multivariate analysis (independent variables: sex, ethnicity, *ABCB1* rs1045642 G/G vs G/A+A/A, *ABCC2* rs2273697 G/G+G/A vs A/A, *COMT* rs4680 G/G+G/A vs A/A, CYP2C19 UM ultra-rapid metabolizer (UM) vs rapid metabolizers (RM) + normal metabolizers (NM) + intermediate metabolizers (IM) + poor metabolizers (PM), NAT2 RA vs. SA, SLC22A1*3, SLC22A1*5, *UGT1A6* rs7592281 G/G vs. G/T).

Table 5 shows the significant associations between genotypes and phenotypes and the pharmacokinetic parameters of rasagiline. For *ABCB1* rs1045642, individuals with G/A+A/A genotypes presented higher AUC_0-∞_/DW than individuals with the G/G genotype (β = 0.144, R^2^= 0.154, *p* = 0.012) and lower V_d_/F and Cl/F (*p* = 0.018; β = −0.217, R^2^= 0.352, *p* = 0.001; and β = −0.144, R^2^= 0.154, *p* = 0.012, respectively). Subjects with the *ABCC2* rs2273697 A/A genotype presented lower t_max_ compared to volunteers with G/G+G/A genotypes (*p* = 0.005; β = −0.775, R^2^= 0.214, *p* = 0.001). Volunteers with the *SLC22A1* *1/*5 (or rs34059508 G/A) genotype had lower C_max_/DW and higher t_max_ (*p* = 0.022; β = −0.616, R^2^= 0.157, *p* = 0.003; and β = 0.476, R^2^= 0.214, *p* = 0.018, respectively) than those with the *1/*1 (or rs34059508 G/G) diplotype. Subjects with the *1/*3 (or rs12208357 C/T) genotype for *SLC22A1* showed lower t_max_ than subjects with the *1/*1 (or rs12208357 C/C) genotype (*p* = 0.038; β = −0.311, R^2^= 0.214, *p* = 0.032). CYP2C19 intermediate metabolizers (IMs) presented higher V_d_/F with respect to poor metabolizers (PM) (*p* = 0.044). Individuals with the SA phenotype for NAT2 presented a significantly higher C_max_/DW value than those with the RA phenotype (*p* = 0.018; β = 0.347, R^2^= 0.05, *p* = 0.018). Individuals with the *UGT1A6* rs7592281 G/T genotype presented higher t_max_ (*p* = 0.021; coefficient β = 0.338, R^2^= 0.214, *p* = 0.029) than those with the G/G genotype. Finally, subjects with the *COMT* rs4680 A/A genotype presented lower AUC_0-∞_/DW and C_max_/DW with respect to subjects with G/A+G/G genotypes (*p* = 0.018; β = −0.170, R^2^= 0.154, *p* = 0.008; and *p* = 0.012; β = −0.197, R^2^= 0.157, *p* = 0.035, respectively) and higher V_d_/F and Cl/F (β = 0.165, R^2^= 0.352, *p* = 0.027; and *p* = 0.018; β = 0.170, R^2^= 0.154, *p* = 0.008, respectively) (Table 5). After the Bonferroni correction for multiple comparisons the following associations were considered statistically significant: *ABCB1* rs1045642 and V_d_/F; *ABCC2* rs2273697 and t_max_; SLC22A1*5 and C_max_/DW (all *p* < 0.001, the corrected threshold for assuming significant associations) (Figure 1). The Vd/F, t_max_ and C_max_/DW were subsequently analyzed (as dependent variables) with three independent GLMs, where the three variants (*ABCB1* rs1045642, *ABCC2* rs2273697 and SLC22A1*5) were included as independent variables. No interaction between the variants was observed for any GLM model (i.e., they can be considered independent predictors).

Only one AE (headache) was causally related to rasagiline intake and therefore considered an ADR. Therefore, statistical relationships between rasagiline safety and pharmacogenetic biomarkers could not be evaluated. This volunteer presented the following genotypes: GG for *ABCB1* rs1045642, GG for *ABCC2* rs2273697, GG for *UGT1A6* rs7592281 and GG for *COMT* rs4680; *1/*1 for *SLC22A1*; IM for CYP2C19 and SA for NAT2. His AUC_0-∞_/DW was 197.49 h*ng*kg/mL*mg and his C_max_/DW was 339.71 ng*kg/mL*mg (i.e., lower compared to the mean observed values).

## 4. Discussion

Rasagiline is prescribed for PD management, which is a chronic disease with no cure. Therefore, it is essential to investigate possible biomarkers that can affect rasagiline bioavailability and the risk for ADRs. In this work, we evaluated the impact of demographic and pharmacogenetic biomarkers on drug’s bioavailability. In congruence with the literature, men presented greater height, weight and BMI than women [25]. The younger age of the Caucasian volunteers can be explained by the fact that they are often interns or students at the Hospital Universitario de La Princesa who discover the possibility of participating in the clinical trial while on rotation. Males presented a higher C_max_/DW than females. This finding is consistent with the reported higher absorption rate in the small intestine in men due to higher gastric motility compared to women [26,27]. In addition, rasagiline is a basic molecule, thus it is better absorbed in the duodenum [28], which reinforces this theory. Women, meanwhile, have a higher percentage of body fat [29] which may affect the V_d_ of drugs depending on their lipophilicity. Rasagiline is a poorly water-soluble compound [28], which likely explains the higher V_d_/FW observed in women. 

It is known that CYP1A2 activity alters rasagiline bioavailability; its plasmatic levels vary when administered with CYP1A2 inhibitors or inducers; a dose reduction is advised in the drug label in case of combination with CYP1A2 inhibitors such as ciprofloxacin [6]. Tobacco is one of the environmental factors that causes an increase of CYP1A2 activity, especially in CYP1A2*1F carriers [30]. However, this is the first study to analyze the impact of the most prevalent *CYP1A2* alleles on rasagiline’s metabolism. In this study, no significant findings were observed in this regard despite performing a robust haplotype analysis. Therefore, it seems that *CYP1A2* genotyping is not clinically relevant for rasagiline prescription. Two possible explanations are that (a) haplotypes or alleles have no effect on the enzymatic capacity of the protein or (b) the enzyme is inducible and compensates for the reduction of function with increased expression [31]. Nevertheless, Table 1 provides a useful tool for further research involving *CYP1A2* that requires accurate allele definition according to the current PharmVAR standard. However, it is important to mention that the current version of PharmVAR *CYP1A2* entry is old, and many of the indexed alleles have limited evidence. That is, they are not unequivocally defined based on sequencing data. Consequently, another explanation raises: if alleles are not properly defined, it is understandable that a particular function may not be inferred. Indeed, three volunteers presented haplotypes that did not match PharmVAR’s allele definitions. Additional efforts are warranted to properly define *CYP1A2* haplotypes, which will be useful to determine allele function, and to evaluate associations between these haplotypes, environmental factors (mainly tobacco) and *CYP1A2* activity. A correct definition of *CYP1A2* will allow to evaluate whether it is a useful biomarker in rasagiline treatment. In the previously cited paper evaluating *CYP1A2* -163C>A as the tagging SNP for *1F [8], authors conclude on the impact of this variant. However, this variant occurs in haplotypes together with other quite prevalent variants, defining alleles other than *1F (the allele containing the −163C>A variant only) (i.e., *1G-*1N) (Table 1). Therefore, it may not be methodologically correct to conclude on the effect of *CYP1A2* −163C>A if this variant is exclusively interrogated. For this reason, alleles may be properly defined and interrogated to conclude on their impact on the enzyme’s function.

Conversely, some suggestive significant relationships were found with the most relevant results obtained involving transporters. Three variants in three genes were observed to be independent predictors of rasagiline pharmacokinetic variability. First, *ABCB1* (ATP-binding cassette sub-family B member 1) encodes the P-glycoprotein (P-gp), an efflux transporter that acts as an ATP-dependent pump, with expression in some clinically relevant regions such as the blood–brain barrier, intestine and liver, preventing drugs from accessing the compartments where they are expressed [32,33]. There are three well-studied variations of the gene with clinical relevance: *ABCB1* rs1128503 (1236C>T), *ABCB1* rs1045642 (3435C>T) and *ABCB1* rs2032582 (2677G>A/T) [34]. In this study, individuals carrying *ABCB1* rs1045642 A allele exhibited a higher exposure to rasagiline. This could be due to the reduced function of the transporter resulting in a lower resistance at intestinal level with respect to its absorption, accumulating in the systemic circulation. In addition, this would result in greater permeation in the central nervous system, and the efficacy of rasagiline in these individuals could be greater. Such an increase in the plasma concentration of the drug was observed in studies with neurotoxic xenobiotics and drugs such as dabigatran [35,36]. Therefore, this biomarker could be understood as protective in terms of rasagiline efficacy, but risky in terms of the occurrence of ADR. To our knowledge, this is the first work to date suggesting these associations, whereas a different study suggested that rasagiline might not be an ABCB1 substrate [37]. Considering that the differences in pharmacokinetic parameters are relatively small, additional investigations are required to test its implication on rasagiline transport. Moreover, further studies are necessary to strengthen the relevance of *ABCB1* as a biomarker, since its different locations throughout the body make it difficult to predict the effect of its polymorphisms on bioavailability, efficacy, and safety. 

Secondly, *ABCC2* is another multidrug resistance protein, a transmembrane efflux transporter that is widely expressed in tissue barriers such as intestine, liver, kidney, and brain. In addition, it is significantly involved in the pharmacokinetics of different substrates and genetic variants can alter its function impacting the bioavailability of various drugs [38]. *ABCC2* rs2273697 A/A volunteers exhibited an approximately 50% lower t_max_ value than G allele carriers, which suggests that absorption is more rapid and, consequently, the concentration of rasagiline increases, as occurs in studies with other drugs such as valproate [39]. In addition, a higher AUC_0-∞_/DW was seen in carriers of this allele with respect to noncarriers, although this association did not reach statistical significance probably because of the low sample size. To our knowledge, this is the first work to date suggesting these two associations. Additional research is warranted to clarify their clinical relevance. 

Thirdly, the organic cation transporter 1 (OCT1) is encoded by the *SLC22A1* gene and is responsible for mediating hepatic uptake of these cations, such as metformin, tramadol, or endogenous compounds such as dopamine. Likewise, *2, *3 and *5 are generally considered decreased-function alleles [40]. In this work, a lower C_max_/DW was observed in individuals carrying the *5 allele and a lower t_max_ in *3 allele carriers, which is not consistent with the literature involving drugs other than rasagiline [41,42]. However, a different study found that *SLC22A1* is involved in the transport of selegiline, another MAO-B inhibitor, which reinforces the theory that rasagiline may also be a substrate of this transporter [43]. Nonetheless, additional studies are necessary to clarify the impact of these polymorphisms.

Regarding metabolizing enzymes, a tendency towards a decrease in bioavailability was observed in UM vs PM individuals for CYP2C19. This suggests that this enzyme is involved in the metabolism of rasagiline; consistently, the structurally related drug selegiline, another MAO inhibitor, is majorly metabolized by CYP2C19 and CYP2B6, which reinforces this hypothesis [44]. Similarly, NAT2 is not described as a rasagiline metabolizing enzyme, however, the reduction in the bioavailability of the drug in individuals with the RA vs SA phenotype is noteworthy, which could be associated with a lower efficacy of the treatment. The same applies for *UGT1A6* considering our findings. Finally, the results obtained for *COMT* can be attributed to a type I error, since a heterozygous advantage is observed and a physiological explanation for this finding is not reasonable.

One limitation of the study was that we were unable to analyze the efficacy of rasagiline, and the safety of rasagiline at steady state, because the clinical trials were conducted with healthy volunteers and with a single administration of the normal dose of the drug. Most ADRs to rasagiline will likely occur in patients who have received the drug on a long-term basis. Moreover, an increased sample size could be especially useful in those genes in which genetic variation is less frequent, such as *SLC22A1*, *UGT1A6* or *NAT2*. Nevertheless, a strength of the study is that it was the first to comprehensively analyze the relationship between genetic variants and variability in pharmacokinetic parameters. Thus, it provides a basis for future research to complement and confirm the proposed associations or implications of genetics in their metabolism. Eventually, this type of studies helps implementing pharmacogenetic information in clinical guidelines. In contrast, the lack of previous published studies with which to compare the results prevented us from validating the relevance of the associations. The present work only addressed part of the pharmacogenetic variables that may alter the response to the treatment with rasagiline. It is possible that other pharmacogenetic biomarkers may alter it, such as *MAOB* or dopaminergic receptors polymorphism, which could alter the pharmacodynamics and, consequently, effectiveness of the drug. However, this study was performed in a very controlled environment that allowed us to avoid confounding factors common in clinical research with patients (e.g., smoking, or concomitant medication). However, some of this confounding factors may be determinants of therapy response in patients (e.g., carriers of the CYP1A2*1F allele could be significantly affected in the real life setting if they are smokers).

## 5. Conclusions

*CYP1A2* polymorphism apparently does not affect rasagiline pharmacokinetics, thus it may not be a clinically relevant pharmacogenetic biomarker. However, gene haplotypes need to be defined with an appropriate methodology to confirm this statement. On the contrary, our results suggest that the genetic polymorphism of drug transporters (i.e., of *ABCB1, ABCC2 and SLC22A1*) may explain part of the pharmacokinetic variability of rasagiline. Further research is warranted to confirm the clinical relevance of our findings. 

## Figures and Tables

**Table 1:** Allele definition table for *CYP1A2*.

Alelle	−3860G>A	−739T>G	−729C>T	−163C>A	2499A>T	5347T>C
	rs2069514	rs2069526	rs12720461	rs762551	rs72547516	rs2470890
*1A (normal activity)	G	T	C	C	A	T
*1B						C
*1C (decreased activity)	A					
*1E		G				
*1F (high inducibility)				A		
*1G		G				C
*1J		G		A		
*1K (decreased activity)		G	T	A		
*1L	A			A		C
*1N				A		C
*4 (decreased expression)					T	

Enzyme activity associated with each allele is shown in brackets when known, obtained from PharmVAR. The * refers to standarized star alleles.

**Table 2:** Demographic characteristics of the study population according to sex and ethnicity.

Sex	*n*	Age (Years)	Height (m)	Weight (kg)	BMI (kg/m^2^)
Women	65	25 (IQR = 9)	1.62 ± 0.06	60.06 ± 7.89	22.87 ± 2.72
Men	53	24 (IQR = 10)	1.76 ± 0.06 *	75.18 ± 9.59 *	24.30 ± 2.97 *
Ethnicity					
Caucasian	83	24 (IQR = 7)	1.70 ± 0.09	66.38 ± 10.89	22.91 ± 2.65
Mixed	35	30 (IQR = 15) *	1.64 ± 0.09 *	67.98 ± 12.89	24.96 ± 3.04 *
Total	118	25 (IQR = 9)	1.68 ± 0.09	66.85 ± 11.49	23.52 ± 2.91

* *p* < 0.05 after *t*-test or Kruskal–Wallis test; BMI: body mass index. Results are shown as mean ± SD or median (interquartile range, IQR).

**Table 3:** Pharmacokinetic parameters of rasagiline according to sex and ethnicity.

Sex	*n*	AUC_0-∞_/DW (h*ng*kg/mL*mg)	C_max_/DW (ng*kg/mL*mg)	t_max_ (h)	t_1/2_ (h)	V_d_/F (L/kg)	Cl/F (L/kg*h)
Women	65	254.19 ± 74.50	378.42 ± 156.70	0.48 ± 0.19	2.98 ± 0.80	17.98 ± 6.23	4.30 ± 1.39
Men	53	278.61 ± 73.51	447.91 ± 163.88 *	0.52 ± 0.28	2.31 ± 0.85 *^&^	12.18 ± 3.78 *^&^	3.85 ± 1.08
Ethnicity							
Caucasian	83	258.45 ± 71.81	418.18 ± 169.33	0.47 ± 0.19	2.56 ± 0.84	15.00 ± 5.72	4.18 ± 1.23
Mixed	35	281.05 ± 80.09	389.35 ± 147.25	0.55 ± 0.31	2.95 ± 0.94 *	16.27 ± 6.62	3.92 ± 1.39
Total	118	265.16 ± 74.74	409.63 ± 163.00	0.49 ± 0.24	2.68 ± 0.89	15.38 ± 6.00	4.10 ± 1.28

* *p* < 0.05 after *t*-test; Underlined: *p* < 0.05 after multivariate analysis (independent variables: sex, ethnicity, *ABCB1* rs1045642 G/G vs G/A+A/A, *ABCC2* rs2273697 G/G+G/A vs A/A, *COMT* rs4680 G/G+G/A vs A/A, CYP2C19 ultra-rapid metabolizer (UM) vs rapid metabolizers (RM)+ normal metabolizers (NM)+ intermediate metabolizers (IM)+ poor metabolizers (PM), NAT2 RA vs SA, SLC22A1*3, SLC22A1*5, *UGT1A6* rs7592281 G/G vs G/T); ^&^ *p* < 0.001 or significant after Bonferroni correction for multiple comparisons.

**Table 4:** Pharmacokinetic parameters of rasagiline according to *CYP1A2* diplotypes.

*CYP1A2* Diplotype	*n*	AUC_0-∞_/DW (h*ng*kg/mL*mg)	C_max_/DW (ng*kg/mL*mg	t_max_ (h)	t_1/2_ (h)	V_d_/F (L/kg)	Cl/F (L/kg*h)
*1F/*1F	38	263.28 ± 60.08	403.23 ± 139.11	0.51 ± 0.21	2.69 ± 0.82	15.32 ± 5.34	3.98 ± 0.86
*1B/*1F	36	265.27 ± 80.68	426.5 ± 183.44	0.44 ± 0.19	2.66 ± 0.9	15.24 ± 5.73	4.15 ± 1.41
*1L/*1F	11	285.81 ± 77.84	389.59 ± 138.34	0.64 ± 0.48	2.55 ± 1.07	13.05 ± 4.16	3.82 ± 1.36
*1B/*1B	6	226.81 ± 52.00	411.84 ± 85.38	0.42 ± 0.18	1.87 ± 0.6	11.98 ± 3.33	4.62 ± 1.15
*1L/*1L	6	301.28 ± 89.3	518.16 ± 158.15	0.53 ± 0.17	3.06 ± 0.38	15.94 ± 5.55	3.61 ± 1.23
*1B/*1N	5	256.33 ± 116.11	327.66 ± 320.93	0.6 ± 0.28	3 ± 0.78	19.45 ± 9.55	4.63 ± 2.23
*1J/*1N	5	275.39 ± 91.37	435.74 ± 117.78	0.43 ± 0.09	2.15 ± 0.83	11.59 ± 3.81	3.9 ± 1.02
*1B/*1L	4	275.25 ± 108.77	392.87 ± 133.43	0.46 ± 0.16	3.32 ± 1.62	18.28 ± 7.57	4.11 ± 1.66
*1N/*1K, *1N/*1L, *1N/*1L and *1N/*1G	4	220.53 ± 87.92	355.79 ± 242.1	0.5 ± 0.14	2.94 ± 0.69	22.89 ± 12.33	5.21 ± 2.41
Total	115	265.29 ± 75.63	411.77 ± 164.35	0.49 ± 0.24	2.66 ± 0.89	15.32 ± 6.06	4.11 ± 1.29

Only one volunteer was observed for each of the following genotypes: *1N/*1K, *1N/*1L, *1N/*1L or *1N/*1G, thus they were merged for statistical analysis. Three volunteers had haplotypes other than those defined by PharmVAR and were excluded from the analysis.

**Table 5:** Associations between genotypes and phenotypes and the pharmacokinetic parameters of rasagiline.

Genotype or Phenotype		*n*	AUC_0-∞_/DW (h*ng*kg/mL*mg)	C_max_/DW (ng*kg/mL*mg)	t_max_ (h)	t_1/2_ (h)	V_d_/F (L/kg)	Cl/F (L/kg*h)
*ABCB1* rs1045642	G/G	34	**244.10 ± 63.87 (p_m_ = 0.012)**	359.70 ± 127.40	0.47 ± 0.19	2.83 ± 0.73	**17.59 ± 5.97 (p_u_ = 0.018, p_m_ = 0.001) ^$^**	**4.41 ± 1.31 (p_m_ = 0.012)**
	G/A	61	273.00 ± 83.60	426.48 ± 179.87	0.51 ± 0.27	2.63 ± 1.04	14.71 ± 6.41	4.05 ± 1.38
	A/A	23	275.48 ± 59.65	438.76 ± 152.32	0.50 ± 0.21	2.58 ± 0.63	13.89 ± 3.87	3.79 ± 0.79
*ABCC2* rs2273697	G/G	73	262.04 ± 73.57	401.58 ± 167.92	0.51 ± 0.26	2.71 ± 0.89	15.73 ± 5.94	4.13 ± 1.23
	G/A	42	264.50 ± 74.33	411.51 ± 152.39	0.48 ± 0.19	2.59 ± 0.90	14.95 ± 6.30	4.13 ± 1.36
	A/A	3	350.15 ± 86.34	579.28 ± 133.19	**0.22 ± 0.09 (p_u_ = 0.005, p_m_ = 0.001) ^&^**	3.01 ± 0.49	12.72 ± 2.28	2.99 ± 0.80
SLC22A1*5 (rs34059508)	*1/*1	113	267.52 ± 75.21	415.96 ± 161.85	0.49 ± 0.23	2.70 ± 0.89	15.38 ± 6.07	4.07 ± 1.29
	*1/*5	5	211.83 ± 36.15	**266.58 ± 130.60 (p_u_ = 0.022, p_m_ = 0.003)**	**0.67 ± 0.24 (p_u_ = 0.018)**	2.24 ± 0.71	15.36 ± 4.85	4.83 ± 0.79
SLC22A1*3 (rs12208357)	*1/*1	104	265.80 ± 73.86	401.10 ± 159.35	0.51 ± 0.24	2.71 ± 0.89	15.52 ± 6.05	4.10 ± 1.32
	*1/*3	8	258.87 ± 34.73	473.14 ± 99.07	**0.35 ± 0.11 (p_u_ = 0.038 p_m_ = 0.032)**	2.22 ± 0.81	12.35 ± 3.96	3.93 ± 0.58
CYP2C19	UM	3	205.53 ± 82.32	347.93 ± 233.78	0.61 ± 0.35	1.75 ± 0.67	15.39 ± 12.27	5.49 ± 2.41
	RM	21	274.03 ± 86.22	426.69 ± 180.45	0.41 ± 0.11	2.66 ± 0.85	14.82 ± 6.62	4.09 ± 1.61
	NM	56	267.83 ± 70.38	402.65 ± 142.62	0.53 ± 0.27	2.67 ± 0.91	15.07 ± 5.84	4.02 ± 1.20
	IM	36	258.70 ± 75.90	415.50 ± 185.56	0.46 ± 0.22	2.85 ± 0.84	16.62 ± 5.25	4.16 ± 1.08
	PM	2	302.71 ± 14.98	412.92 ± 55.36	0.75 ± 0.11	1.55 ± 0.41	7.33 ± 1.58	3.31 ± 0.16
NAT2	RA	9	230.53 ± 67.28	315.46 ± 178.72	0.50 ± 0.24	2.72 ± 0.68	17.96 ± 5.87	4.69 ± 1.46
	SA	103	266.53 ± 75.05	**420.47 ± 161.94 (p_u_ = 0.018, p_m_ = 0.018)**	0.49 ± 0.24	2.62 ± 0.83	15.07 ± 6.05	4.08 ± 1.27
*UGT1A6* rs7592281	G/G	111	264.10 ± 73.64	409.07 ± 159.30	0.48 ± 0.22	2.66 ± 0.89	15.25 ± 5.93	4.10 ± 1.23
	G/T	7	281.88 ± 95.76	418.57 ± 229.87	**0.70 ± 0.32 (p_u_ = 0.021, p_m_ = 0.029)**	3.03 ± 0.72	17.43 ± 7.29	4.08 ± 1.96
*COMT* rs4680	G/G	36	253.96 ± 59.13	385.75 ± 140.56	0.52 ± 0.32	2.58 ± 0.68	15.39 ± 5.13	4.14 ± 0.93
	G/A	56	285.72 ± 81.43	450.03 ± 166.19	0.47 ± 0.18	2.71 ± 0.95	14.40 ± 6.06	3.84 ± 1.33
	A/A	26	**236.37 ± 68.34 (p_u_ = 0.018, p_m_ = 0.008)**	**355.69 ± 168.61 (p_u_ = 0.012, p_m_ = 0.035)**	0.50 ± 0.22	2.74 ± 1.00	**17.45 ± 6.66 (pm=0.027)**	**4.61 ± 1.45 (pm=0.008)**
TOTAL		118	265.16 ± 74.74	409.63 ± 163.01	0.49 ± 0.24	2.68 ± 0.89	15.38 ± 6.00	4.10 ± 1.28

UM: ultra-rapid metabolizer. RM: rapid metabolizer. NM: normal metabolizer. IM: intermediate metabolizer. PM: poor metabolizer. SA: slow acetilator. RA: rapid acetilator. p_u_: *p*-value in the univariate analysis, p_m_: *p*-value in the multivariate analysis. Only *p*-values < 0.05 are included in the table. Independent variants included in the multivariate analysis: sex, ethnicity, *ABCB1* rs1045642 G/G vs G/A+A/A, *ABCC2* rs2273697 G/G+G/A vs A/A, *COMT* rs4680 G/G+G/A vs A/A, CYP2C19 UMs vs RMs+NMs+IMs+PMs, NAT2 RAs vs SAs, SLC22A1*3, SLC22A1*5, *UGT1A6* rs7592281 G/G vs G/T; ^&^ *p* < 0.001 or significant after Bonferroni correction for multiple comparisons.

## Data Availability

Data belong to the clinical trials’ sponsors and may be accessible upon reasonable request to the corresponding authors.

## Supplementary Materials

The following supporting information can be downloaded at: https://www.mdpi.com/article/10.3390/pharmaceutics14102001/s1, Table S1. VIPOA2 customized array genes and alleles/variants analyzed in the present work.
