# Supplementary material for: Polymorphism of Drug Transporters, Rather Than Metabolizing Enzymes, Conditions the Pharmacokinetics of Rasagiline
Source: Pharmaceutics. 2022 Sep 21;14(10):2001. doi: 10.3390/pharmaceutics14102001 (PMC9610285; doi:10.3390/pharmaceutics14102001)
Supplement: Supplementary file 1 [file pharmaceutics-14-02001-s001.zip › pharmaceutics-1883739-supplementary.pdf]

Table S1. VIPOA2 customized array genes and alleles/variants analyzed in the present work.

Gene	Allele/Variant	Gene	Allele/Variant	Gene	Allele/Variant
ABCB1	C1236T (rs1128503)	*CYP2C19*	*6 (rs72552267)	*CYP2D6*	*14 (rs5030865)
	C3435T (rs1045642)		*7 (rs72558186)		*15 (rs774671100)
	G2677A (rs2032582)		*8 (rs41291556)		*17 (rs28371706)
	G2677T (rs2032582)		*9 (rs17884712)		*19 (rs72549353)
ABCC2	rs2273697		*17 (rs12248560)		*29 (rs59421388)
	rs3740066		*35 (rs12769205)		*41 (rs28371725)
ABCC3	rs4793665	*CYP2C8*	*2 (rs11572103)		*56B (rs72549347)
	rs9895420		*3 (rs10509681)		*59 (rs79292917)
ABCG2	rs2231137		*3 (rs11572080)	*NAT2*	*6 (rs1799930)
	rs2231142		*4 (rs1058930)		*7 (rs1799931)
	rs7699188	*CYP2C9*	*2 (rs1799853)		*5 (rs1801280)
CES1	rs71647871		*3 (rs1057910)	*SLC22A1*	rs628031
	rs2244613		*5 (rs28371686)		*2 (rs72552763)
	rs8192935		*8 (rs7900194)		*3 (rs12208357)
COMT	rs13306278		*8 (rs9332094)		*5 (rs34059508)
	rs4680		*11 (rs28371685)	*SLC6A2*	rs12708954
	rs4818	*CYP3A4*	*1B (rs2740574)		rs3785143
	rs5993883		*1G (rs2242480)	*SLC22A2*	rs316019
CYP1A1	*2A (rs4646903)		*2 (rs55785340)	*SLC28A3*	rs7853758
	*2B (rs1048943)		*3 (rs4986910)	*SLCO1B1*	rs2306283
	*4 (rs1799814)		*4 (rs55951658)		*2 (rs56101265)
CYP1A2	*1B (rs2470890)		*5 (rs55901263)		*3, *13 (rs56061388)
	*1C (rs2069514)		*6 (rs4646438)		*4, *14 (rs11045819)
	*1E (rs2069526)		*18 (rs28371759)		*5 (rs4149056)
	*1F (rs762551)		*20 (rs67666821)		*6 (rs55901008)
	*1K (rs12720461)		*22 (rs35599367)		*9, *31 (rs59502379)
	*4 (rs72547516)	*CYP3A43*	*2 (rs61469810)		*10, *12 (rs56199088)
CYP1B1	*2 (rs10012)	*CYP3A5*	*3 (rs776746)		*17, *21 (rs4149015)
	*3 (rs1056836)		*6 (rs10264272)	*UGT1A1*	rs8330
	*4 (rs1800440)		*7 (rs41303343)		*6 (rs4148323)
CYP2A6	rs28399433	*CYP2D6*	*2 (rs16947)		*80 (rs887829)
CYP2B6	*4, *7, *16 (rs2279343)		*2, *8, *10 (rs1135840)	*UGT1A4*	rs2011425
	*5, *7 (rs3211371)		*3 (rs35742686)	*UGT1A6*	rs7592281
	*6, *7, *9 (rs3745274)		*4 (rs3892097)		*3A (rs10445704)
	*16, *18 (rs28399499)		*6 (rs5030655)	*UGT1A8*	*2 (rs1042597)
	*22 (rs34223104)		*7 (rs5030867)	*UGT1A9*	rs10929302
CYP2C19	*2 (rs4244285)		*8 (rs5030865)	*UGT2B7*	*2(rs7668258)
	*3 (rs4986893)		*9 (rs5030656)	*UGT2B10*	rs61750900
	*4 (rs28399504)		*10 (rs1065852)	*UGT2B15*	rs1902023
	*5 (rs56337013)		*12 (rs5030862)	*UGT1A3-4*	rs2008584

Alleles are shown along with tagging single nucleotide polymorphisms (SNPs) or variants; however, one variant may appear in several alleles.
